# Composite Hydrogel of Methacrylated Hyaluronic Acid and Fragmented Polycaprolactone Nanofiber for Osteogenic Differentiation of Adipose-Derived Stem Cells

**DOI:** 10.3390/pharmaceutics12090902

**Published:** 2020-09-22

**Authors:** Madhumita Patel, Won-Gun Koh

**Affiliations:** Department of Chemical and Biomolecular Engineering, Yonsei University, 50 Yonsei-ro, Seodaemoon-gu, Seoul 03722, Korea; madhurk29@gmail.com

**Keywords:** hydrogel composite, fragmented nanofiber, methacrylated hyaluronic acid, osteogenic differentiation

## Abstract

Composite hydrogels with electrospun nanofibers (NFs) have recently been used to mimic the native extracellular matrix. In this study, composite hydrogels of methacrylated hyaluronic acid containing fragmented polycaprolactone NFs were used for bone tissue engineering. The composite (NF/hydrogel) was crosslinked under ultraviolet (UV) light. The incorporation of fragmented polycaprolactone NFs increased the compression modulus from 1762.5 to 3122.5 Pa. Subsequently, adipose-derived stem cells incorporated into the composite hydrogel exhibited a more stretched and elongated morphology and osteogenic differentiation in the absence of external factors. The mRNA expressions of osteogenic biomarkers, including collagen 1 (Col1), alkaline phosphatase, and runt-related transcription factor 2, were 3–5-fold higher in the composite hydrogel than in the hydrogel alone. In addition, results of the protein expression of Col1 and alizarin red staining confirmed osteogenic differentiation. These findings suggest that our composite hydrogel provides a suitable microenvironment for bone tissue engineering.

## 1. Introduction

Past research has shown that hydrogel-based scaffolding is suitable for use in regenerative engineering, mostly as an artificial extracellular matrix (ECM) [1,2]. Hydrogels mimic the soft and flexible structure as well as high-water content of native ECM. Hydrogel-based scaffolding plays a crucial role for 3D cell culture owing to its similar physical and chemical properties to living tissues, and is commonly used for cellular scaffolds by surface attachment or encapsulation to produce a variety of biological outcomes. It can fulfill the requirement of three dimensional regenerative tissue where cells can interact with their surroundings. Hydrogels have been studied for various biomedical purposes such as drug delivery, tissue engineering, 3D cell culture and postsurgical adhesion prevention [3,4,5,6,7,8].

Hydrogels can be prepared from various synthetic and natural polymers [9]. However, synthetic hydrogel have been extensively studied due to their wide variability and easily tunable properties. Hydrogels can be prepared in response to various stimuli including temperature, pH, ionic strength and light [10]. Among others, photo-crosslinking is a simple process to synthesize three-dimensional hydrogel networks. Photo-initiators in a photo-crosslinkable polymer solution can assist in the crosslinking of the polymer under short-term exposure to ultraviolet (UV) light. During the exposure process, the photo-initiator decomposes and produces free radicals, which can induce the crosslinking of methacrylate or acrylate groups [11]. The process of using photo-crosslinking polymers to fabricate three-dimensional hydrogels is rapid and cost-effective [2], and allows for the control of hydrogel size and shape. In addition, the physicochemical properties of hydrogels, such as mechanical properties, degradation rates and swelling ratios, can be adjusted by controlling the UV intensity, photo-initiator or monomer concentrations, crosslinking time and molecular weight [12,13,14]. As such, photo-crosslinked hydrogels have been used for a variety of biomedical applications, such as drug delivery, tissue engineering and biosensing [15,16,17].

Hyaluronic acid (HA), a glycosaminoglycan composed of repeating units of d-*N*-acetylglucosamine and d-glucuronic acid [18], is a nonadhesive, nonthrombogenic and nonimmunogenic polymer [19,20,21]. HA is a major ECM component found in cardiovascular tissue, cartilage and synovia, and has yielded promising results for bone tissue engineering applications [22,23,24,25]. Recently, the composite system of HA was used to promote osteogenic differentiation. For example, a composite of HA with graphene oxide and chitosan enhanced bone tissue regeneration, both in in vitro and in vivo [26]. Similarly, a hydroxyapatite-embedded, hyaluronic acid-alginate hydrogel system was used to repair bone defects in rat [27]. Another hybrid hydrogel of arginine-based unsaturated poly (ester amide) and methacrylated hyaluronic acid was used to enhance osteogenic differentiation in osteoblast cells [28]. In another study, HA-analogue polymers with saccharide and carboxylate units mimicked the function of HA and were able to induce stem cells for both osteogenic and chondrogenic differentiation [29]. HA composite with poly(lactic-*co*-glycolic acid) is another promising scaffold for bone regeneration [30].

Modification of the methacrylate group in HA with the addition of a photo-initiator and UV exposure leads to photo-polymerization [31]. Methacrylated HA (MeHA) hydrogels exhibit lower degradation rates and good biocompatibility with 3T3 cells and chondrocytes [32]. Although MeHA hydrogels are suitable candidates for biological applications, their nonadhesive nature limits their use in biomedical applications where cell adhesion and spreading are involved. Cell adhesion and spreading is important to determine the fate of stem cells. For example, it has been reported that spread cells and round cells tended to differentiate into osteoblasts and adipocytes from mesenchymal stem cells, respectively [33]. Various attempts have been made to overcome the limitations of cell attachment and spreading in HA hydrogels. For example, the addition of gelatin methacrylate to HA has been used to promote endothelial cell stretching [34,35], while adipose-derived stem cell (ADSC) spreading was improved with the crosslinking of thiolated heparin (Hep-SH) with HA [36].

Hybrid hydrogel fabrication has become a popular approach to improve the biological properties of biomaterials [37]. Both nanofibers and hydrogels have been widely used as scaffolds due to their unique properties. Electrospun polymeric nanofibers are structurally similar to native ECM, with both having high surface-area-to-volume ratios and desirable mechanical properties. However, despite numerous efforts to incorporate nanofibers into hydrogels, only a few reports have been published on fragmented nanofibers in hydrogel scaffolds [38,39]. For example, small fragmented poly(3-caprolactone-*co*-d,l-lactide) (PCL:DLLA) nanofibers and collagen dispersed in a hyaluronan–methylcellulose (HAMC) hydrogel were used as a cell delivery carrier system for the treatment of spinal cord injury [38], while a composite hydrogel consisting of alginate graft-hyaluronate (Alg-g-HA) and poly(lactic acid) (PLA) fibers was used for cartilage tissue regeneration [39].

In this study, we fabricated a fragmented nanofiber–hydrogel composite. Polycaprolactone (PCL) fibers were successfully hydrolyzed into fragmented nanofibers and dispersed in a MeHA solution to generate an ECM-mimicking environment for bone tissue engineering. After the preparation of a composite consisting of the fragmented PCL nanofibers and HA hydrogel, mechanical properties and differences in cellular properties, such as cell spreading and adhesion, were compared with those of bare HA hydrogel without fragmented PCL nanofibers. We also evaluated the osteogenic gene expression of cells in this environment.

## 2. Materials and Methods

### 2.1. Materials

MeHA and the photo-initiator 2-hydroxy-4′-(hydroxyethoxy) 2-methylpropiophenone (Irgacure 2959) were purchased from Blafar (Dublin, Ireland). PCL (MW 80,000), 2,2,2-trifluoroethanol (TFE), and sodium hydroxide (NaOH) were purchased from Sigma-Aldrich (Milwaukee, WI, USA).

### 2.2. Preparation of Fragmented PCL Nanofibers

Fragmented PCL nanofibers were obtained by hydrolyzing electrospun PCL mesh in NaOH solution [40]. The fiber mesh was prepared from 20 wt.% PCL dissolved in TFE. The polymer solution was filled into a syringe and ejected through a 23 G needle. The flow rate and voltage of electrospinning were 0.5 mL/h and 8.0 kV, respectively. The distance between the syringe needle and the aluminum substrate was set to 15 cm. The nanofibers were removed from the substrate with ethanol, washed with distilled water and then hydrolyzed in NaOH (1 M) solution for 10 h. The hydrolyzed fibers were then washed with distilled water to separate the salt. Then, the fragmented nanofiber morphology was observed under SEM and the length and diameter of the nanofibers were measured manually from SEM images using the line tool in Image J program (v 1.50, National Institutes of Health, Bethesda, MD, USA).

### 2.3. Preparation of Nanofiber–Hydrogel Composite

An MeHA-based hydrogel was produced according to the manufacturer’s instructions. Briefly, MeHA was dissolved in phosphate-buffered saline (PBS) at a concentration of 1% (*w*/*v*); then, 0.5% (*w*/*v*) photo-initiator solution was added to the MeHA solution. The fragmented nanofibers were mixed into the solution at 10 wt.% and 20 wt.% after sterilization under UV light. The polymer solution was photo-polymerized for 5 min at 365 nm with 300 mW/cm^2^ UV light (EFOS Ultracure 100SS Plus UV spot lamp, Mississauga, Ontario, Canada). A hydrogel disc was prepared with 100 µL of the polymer solution using a mold with a 3 mm height and a 8 mm diameter. The morphology of the resultant hydrogel was confirmed with scanning electron microscopy (SEM) after the material was freeze-dried.

### 2.4. Rheological Properties of Composite

The hydrogel disc obtained after photo-polymerization was subjected to rheological analysis using an MCR-302 rheometer (Anton Paar, Graz, Austria). The storage modulus (G’) and loss modulus (G”) were monitored under three conditions at room temperature: hydrogel alone and with either 10 wt.% or 20 wt.% fragmented nanofibers.

### 2.5. Cell Culture and Encapsulation

In this study, we used ADSC for osteogenic differentiation, as it is considered the most promising candidate for bone tissue engineering [41]. ADSCs were cultured in Dulbecco’s modified Eagle’s medium with low glucose (DMEM L/G, Hyclone, Logan, UT, USA) supplemented with 10% fetal bovine serum (FBS, CellSera, Rutherford, NSW, Australia) and 1% penicillin-streptomycin (P/S, Hyclone, Logan, UT, USA). After cell confluency, the cells were collected using trypsin (Gibco, Grand Island, NY, USA) and used for cellular experiments. ADSCs were encapsulated in the three types of MeHA hydrogel (i.e., hydrogel alone and hydrogel with either 10 wt.% or 20 wt.% fragmented nanofibers) at a density of 1.0 × 10^6^ cells/mL. Hydrogel discs were prepared using the mold and polymerized under UV at 365 nm and 300 mW/cm^2^ for 5 min. The hydrogel discs were moved to a culture plate and the cells cultured in growth media. 

### 2.6. Cell Viability Assays

Cell proliferation was investigated using Cell-Counting Kit-8 (CCK-8) (Dojindo, Kumamoto, Japan). For this assay, cells encapsulated in hydrogel and composite hydrogel discs were prepared as described earlier. Following cell growth, the medium was replaced with CCK-8 solution, followed by incubation for 3 h in an incubator. After incubation, the absorbance at 450 nm was recorded using a microplate reader (VersaMax; Molecular Devices, Sunnyvale, CA, USA).

### 2.7. Live/Dead Assays

Live/dead assays (Invitrogen, Carlsbad, CA, USA) were conducted to evaluate the viability of the encapsulated ADSCs. The cells in the bare hydrogel and composite hydrogel were incubated with a solution of calcein AM (2 μM) and ethidium (4 μM) for 10 min. Cells were evaluated after 10 days of culture, and fluorescence microscopy (IX53; Olympus Corp., Tokyo, Japan) images were captured for each sample.

### 2.8. Morphology of Encapsulated Cells

To observe the morphology of cells encapsulated in the hydrogel, the cells were stained with phalloidin (Invitrogen, Carlsbad, CA, USA) after three days of culture. The cells were fixed with 4% paraformaldehyde for 10 min and then washed with PBS three times. The cells were permeabilized using 0.1% Triton X-100 in PBS for 10 min. Phalloidin solution was prepared according to the manufacturer’s instructions, and reacted with the cells for 60 min. After rinsing with PBS, the samples were stained with 4′,6-diamidino-2-phenylindole (DAPI) (Molecular Probes, Eugene, OR, USA) to observe the nuclei. Images were then taken under a fluorescence microscope (IX53, Olympus Corp., Tokyo, Japan). Additionally, we analyzed the cell parameters and degree of stretching (Ds) by using Image J program. The cell parameters were calculated by measuring the length of cell membrane (*n* > 5), while Ds was calculated by dividing the long axis by the short axis.

### 2.9. Real-time Polymerase Chain Reaction (RT-PCR) Analysis

An RNA extraction kit (TaKaRa MiniBEST Universal RNA Extraction Kit; TaKaRa, Tokyo, Japan) was used to extract mRNA after 0, 3, 7, and 10 days of culture from all three hydrogel types following the manufacturer’s instructions. The extracted mRNA was collected with RNase-free water, and the concentration was measured using a NanoDrop 1000 spectrophotometer (Thermo Scientific, Willington, DE, USA). Prior to PCR analysis, cDNA was synthesized using a ReverTra Ace® qPCR RT kit (Toyobo, Osaka, Japan). RT-PCR was conducted using a CFX 96 system with SYBR Green Supermix. The relative gene expression level was calculated as 2^−ΔΔCt^, with the target gene expression normalized as ΔΔCt = (Gene A − GAPDH) _t_ − (Gene A − GAPDH) _t0_, with t_0_ indicating day 0. The levels of the osteogenic differentiation biomarkers collagen type I (COL1), alkaline phosphatase (ALP), and runt-related transcription factor 2 (RUNX2) were measured at 0, 3, 7, and 10 days. The primer sequences for COL1, ALP, RUNX2 and glyceraldehyde-3-phosphate-dehydrogenase (GAPDH) are shown in Table 1.

### 2.10. Immunocytochemistry

The immunocytochemistry of the samples was analyzed after 10 days of culture. COL1 (Abcam, Cambridge, UK) protein expression was evaluated to confirm osteogenic differentiation. The samples were fixed in 4% paraformaldehyde and permeabilized with Triton (0.1%) for 10 min each. Subsequently, the samples were incubated with a primary antibody for 1 h, followed by blocking with bovine serum albumin (BSA, Sigma, Saint Louis, MO, USA). The samples were then stained with a secondary antibody (FITC-conjugated AffiniPure Donkey Anti-mouse IgG, Jackson ImmunoResearch, Baltimore, PA, USA) after being rinsed with PBS three times. The nucleus was stained using DAPI. Fluorescent images were obtained using a fluorescence microscope with Olympus cellSens software (Entry version, Olympus Corp, Tokyo, Japan).

### 2.11. Alizarin Red Staining

Alizarin red S staining (ARS) was used to evaluate calcium deposition. For this, 2% ARS (Sigma, St. Louis, MO, USA) solution was prepared in water, with the pH adjusted to 4.3. The samples were incubated in this solution for 30 min, followed by fixation with 4% paraformaldehyde for 10 min. Excess dye was removed by washing the samples with distilled water. Images were then captured under a phase microscope.

### 2.12. Statistics Analysis

Statistical significance were analyzed using one-way ANOVA with Tukey tests, in which * and ** indicate *p* < 0.05 and *p* < 0.01, respectively.

## 3. Results

### 3.1. Physical Characterization of PCL Nanofibers

**Figure 1:**
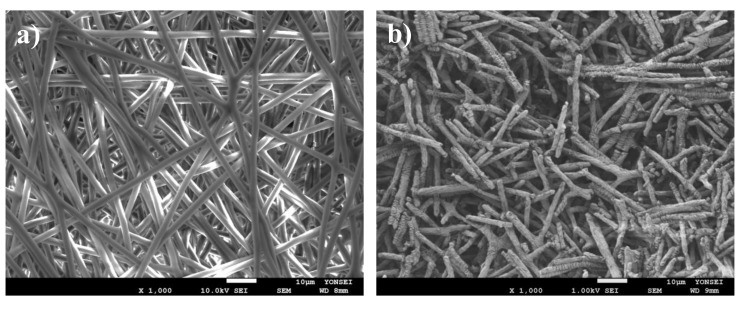
SEM images of (**a**) electrospun PCL nanofibers and (**b**) fragmented PCL nanofibers.

The morphology of the fabricated PCL nanofibers was evaluated using SEM and found to be continuous, uniform and random (Figure 1a). The hydrolyzation of the PCL nanofiber mesh produced short nanofiber fragments (Figure 1b). The average length and diameter of the fragmented nanofibers were 12.34 ± 1.50 µm and 1.8 ± 0.21 µm, respectively [42,43]. Although fragmented nanofibers have been prepared using various methods in past research, hydrolyzation has an advantage over other methods in terms of process efficiency and the uniformity of the size of the fragmented nanofibers. For example, the cryogenic milling method is limited by the elasticity of the fibers [44], while the grinding and milling method lacks fiber length control [45,46]. Similarly, though it affords better fiber length control, the liquid nitrogen method is inefficient [47].

### 3.2. Morphology of Hydrogel Scaffolds

**Figure 2:**
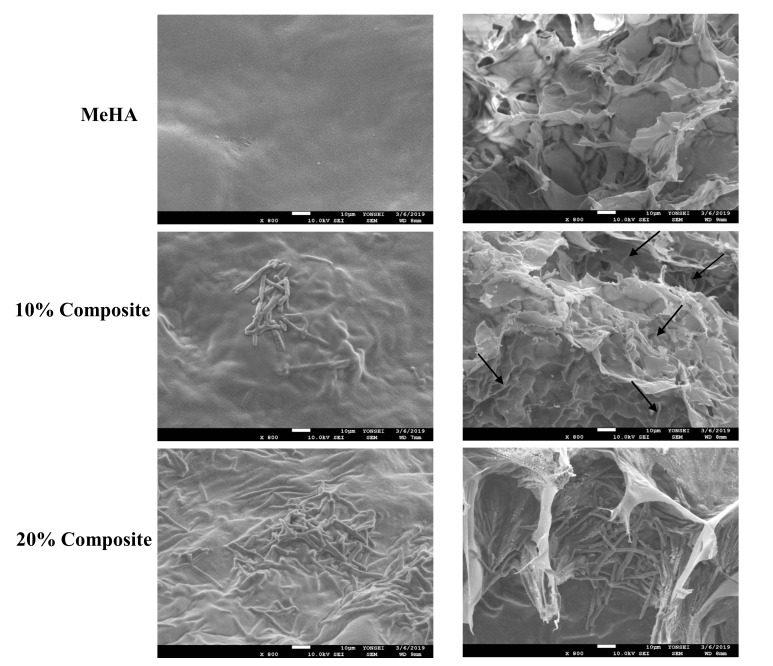
SEM of MeHA hydrogel, 10% and 20% nanofiber–hydrogel composites. (**left**: surface of the scaffold, **right**: cross sectioned of the scaffold). Fibers are visible in the cross section area (black arrow heads).

The nanofiber–hydrogel composite was evaluated using SEM images (Figure 2). The image on the right is a cross-sectional depiction of the composite scaffold and hydrogel alone. The SEM images also confirmed that the nanofibers were homogeneously distributed throughout the hydrogel.

### 3.3. Mechanical Properties of Composite Hydrogel

**Figure 3:**
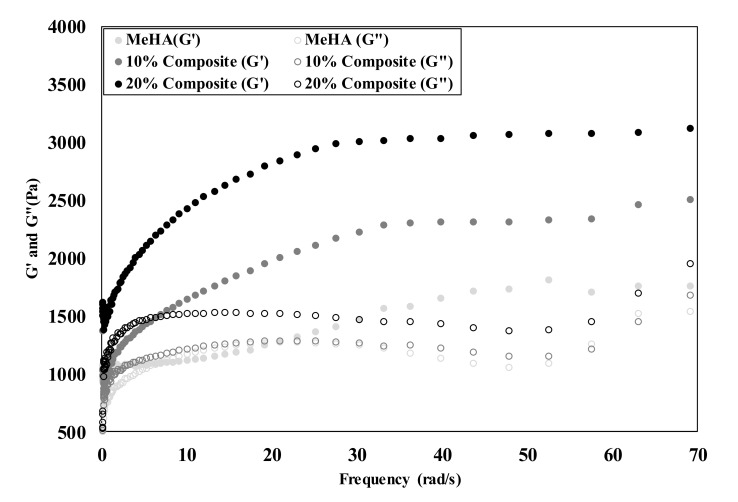
Mechanical properties of MeHA hydrogel, 10% and 20% nanofiber–hydrogel composites. The storage modulus (G′) and loss modulus (G″) under different angular frequencies.

The mechanical properties of a hydrogel significantly affect cell behavior. We evaluated the storage modulus in the presence and absence of nanofibers in the hydrogel (Figure 3). The compressive modulus was found to be 2502.6 ± 73.6 Pa and 3122.5 ± 43.7 Pa for the 10% and 20% nanofiber–hydrogel composites, respectively, while the modulus for the hydrogel without nanofibers was measured at 1762.5 ± 48.5 Pa. The modulus was thus higher with an increase in the proportion of nanofibers. These observations are similar to those reported for other fiber–PEGDA composite hydrogel studies [48]. The findings indicate that the incorporation of fragmented nanofibers can enhance the mechanical properties of pure hydrogels, with the modulus of the composite hydrogels in this study being amenable to bone tissue engineering [49]. Scaffold stiffness plays a key role in stem cell differentiation, with stiffer substrates directing stem cells toward osteogenesis. A scaffold with a stiffness of 1.5 kPa or higher induces greater osteogenic differentiation in the absence of external factors [50].

### 3.4. Cell Viability

**Figure 4:**
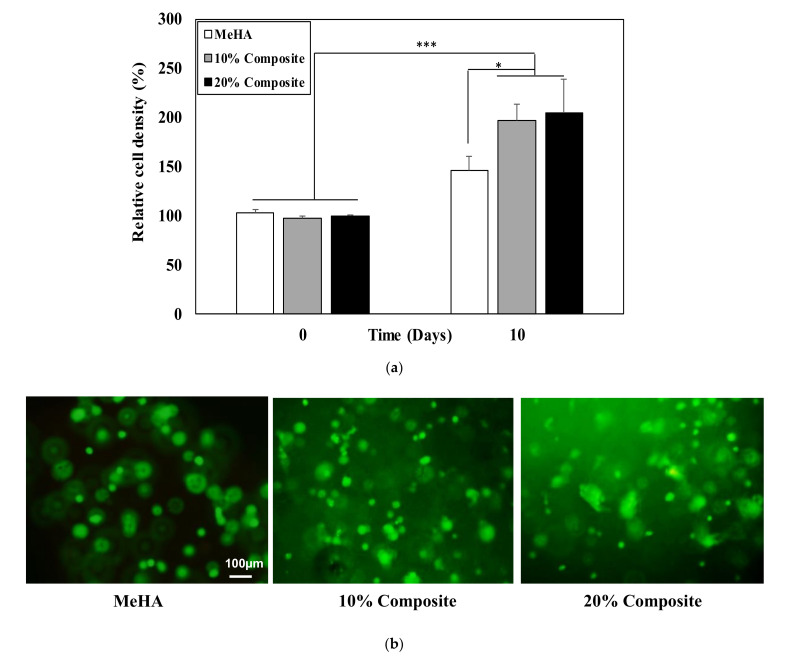
Viability of encapsulated ADSC obtained by (**a**) CCK assay and (**b**) Live/Dead fluorescence assay after 10 days in MeHA hydrogel, 10% and 20% nanofiber–hydrogel composites. The data were analyzed by one-way ANOVA, where * and *** indicate *p* < 0.05 and *p* < 0.001, respectively; *n* = 3.

Cell viability in the composite hydrogels and the hydrogel alone was determined using CCK assays after 1 and 10 days (Figure 4a). The cells in the composite hydrogels showed higher cell proliferation compared with hydrogel alone, with the 20% nanofiber–hydrogel composite producing the highest cell proliferation rate after 10 days. The encapsulated cells were alive after 10 days in all three hydrogels, as revealed by calcein AM staining (Figure 4b).

### 3.5. Cell Morphology

**Figure 5:**
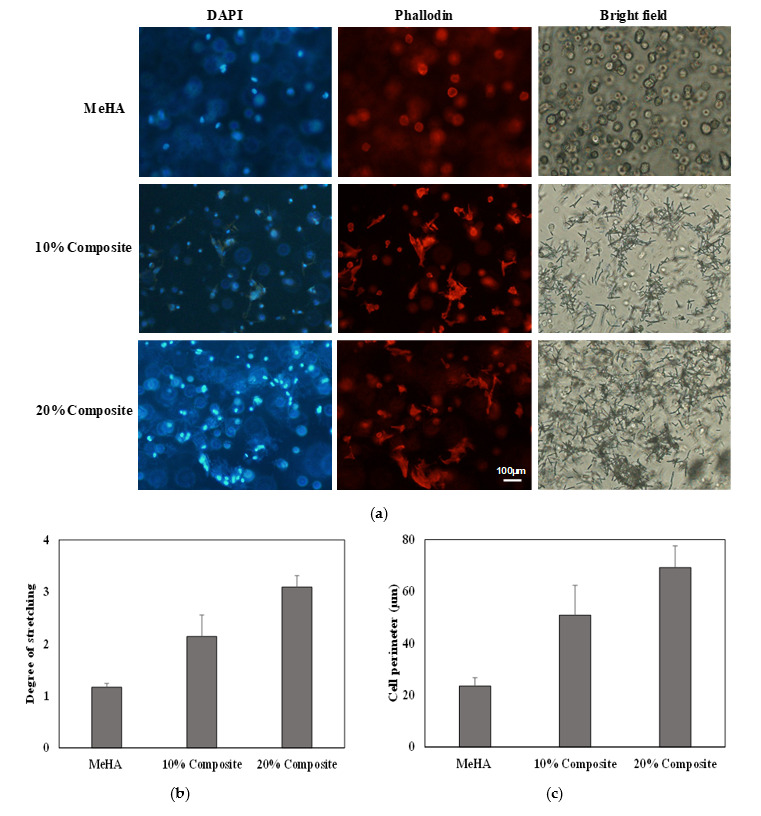
Morphology of encapsulated ADSC in (**a**) MeHA hydrogel, 10% and 20% nanofiber–hydrogel composites after 3d. Cells stained with Phalloidin (red) and DAPI (blue) for visualizing actin and nuclei, respectively. (Scale bar = 100 µm.) (**b**) The degree of stretching and (**c**) the perimeters of cell calculated by image J program. More than five cells were counted and analyzed.

We evaluated the morphology of the ADSCs in the composite scaffolds and hydrogel alone after three days of cell growth. The cell filaments and nuclei were stained with phalloidin and DAPI, respectively. The cells exhibited a more stretched and elongated morphology in both the 10% and 20% nanofiber–hydrogel composite, whereas the cells in hydrogel alone exhibited a round morphology (Figure 5a). The 20% composite in particular yielded well-defined, stretched and elongated cells. Cell adhesion is associated with actin filaments, which suggests that the cells encapsulated in the 20% nanofiber–hydrogel composite provided more surface area for adhesion. Lee et al. reported similar results for a nanofiber–hydrogel composite [40]. The hydrogel alone did not induce cell adhesion due to the nonadhesiveness of HA. The addition of fragmented nanofibers provided attachment sites for encapsulated cells, and consequently, the number of stretched cells increased. We quantified the degree of cell stretching (Figure 5b) and cell perimeter (Figure 5c) in all three conditions. The result shows that both degree of cell stretching and cell parameters increased with the nanofiber content. The cell degree of stretching and parameters showed 2.7-fold and 3-fold increases respectively in the 20% nanofiber–hydrogel composite compared to native hydrogel.

### 3.6. Osteogenic Differentiation of ADSCs in Composite Hydrogel

**Figure 6:**
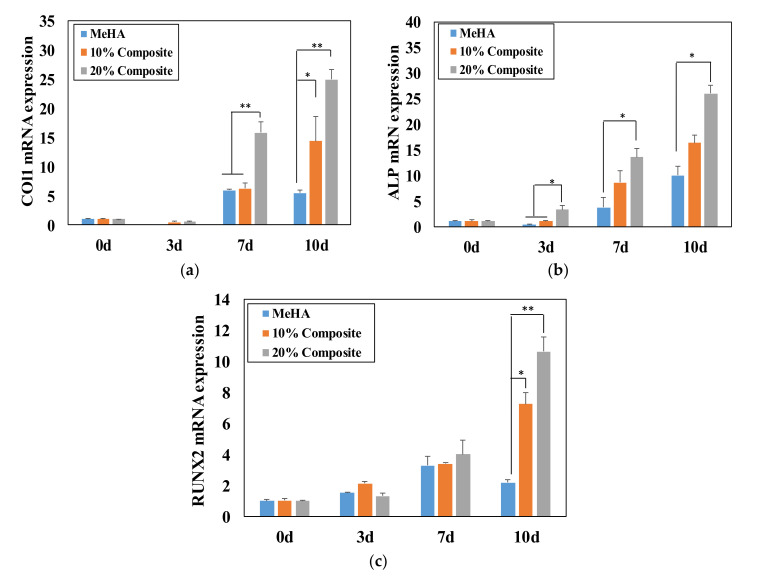
mRNA expression for osteogenic genes of (**a**) Col1, (**b**) ALP and (**c**) RUNX2 of the cells incubated for 0, 3, 7 and 10 days. The expression level of genes was analyzed by real-time qPCR and normalized by GAPDH and on day 0. The data were analyzed by one-way ANOVA, where * and ** indicate *p* < 0.05 and *p* < 0.01, respectively; *n* = 3.

Further, we examined the effect of incorporating fragmented nanofibers on osteogenic differentiation [33]. The mRNA levels of osteogenic genes measured using PCR are presented in Figure 6. The cells encapsulated in the composite hydrogels showed higher gene expression than those in hydrogel alone. In particular, osteogenic biomarkers COL1, ALP and RUNX2 were determined at the mRNA level. COL1 expression increased 3- and 5-fold after 10 days of incubation in the 10% and 20% nanofiber–hydrogel composites, respectively, relative to hydrogel alone, and 3-fold in the 20% composite after seven days (Figure 6a). Variation in the expression of COL1, one of the major proteins in bone, is related to the risk of osteoporosis. COL1 is associated with middle-stage differentiation and is typically observed after seven days of incubation [51].

The mRNA expression levels of ALP increased significantly after three days of incubation. In particular, ALP mRNA levels in the 20% n nanofiber–hydrogel composite increased 3-, 3-, and 2.6-fold after 3, 7 and 10 days, respectively, compared with hydrogel alone (Figure 6b). ALP is an early-stage marker of osteogenesis [52], and is involved in bone mineralization.

RUNX2 is one of the most important osteogenic transcription factors, playing a crucial role in early osteogenic differentiation [53]. It is also essential for proper skeletal development. RUNX2 mRNA levels increased 2- and 5-fold after 10 days of incubation in the 10% and 20% composites, respectively, compared with hydrogel alone (Figure 6c). Hydrogel alone also increased the mRNA expression of COL1, ALP and RUNX2 over the 10-day incubation period. The cell culture conditions in this study all contained a native ECM in the form of HA, which induced osteogenic differentiation and influenced the cell shape in the presence of nanofibers in the hydrogels.

**Figure 7:**
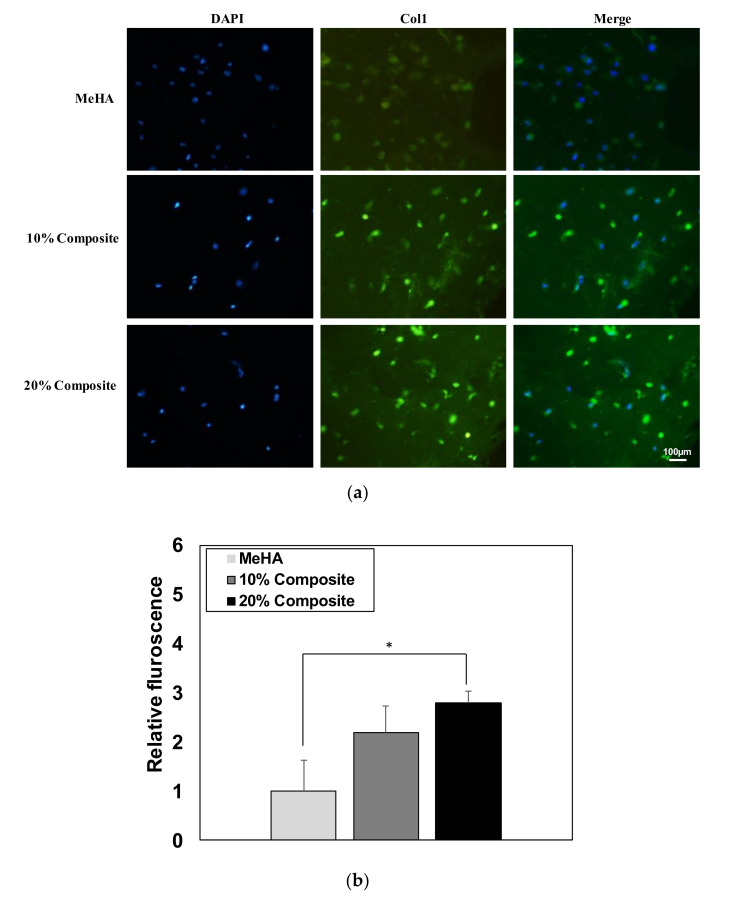
(**a**) Immunofluroscence images of encapsulated ADSC with Col1 protein expression. Cells stained green and nuclei stained with blue by DAPI. (**b**) Semi-quantitative analysis of fluorescence intensity. Data were analyzed by one-way ANOVA, where * indicate *p* < 0.05, *n* = 3.

The incubated ADSCs were also analyzed using immunofluorescence staining for COL1 to investigate osteogenic protein expression levels. The osteogenic biomarkers were stained in green and the nuclei were stained in blue using DAPI. The images confirmed that the fluorescence intensity was higher in the composite hydrogels (Figure 7a). A semiquantitative analysis (Figure 7b) of the fluorescence was calculated by multiplying the number of stained cells by the fluorescence intensity using Image J program. These immunofluorescence findings thus corroborated the mRNA expression results.

**Figure 8:**
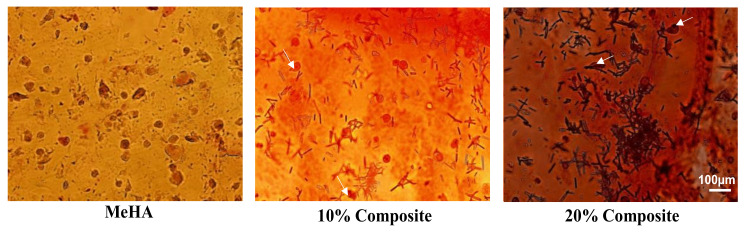
Alizarin red assay of encapsulated ADSC in MeHA hydrogel, 10% and 20% nanofiber–hydrogel composites. (Scale bar = 100 um.).

Additionally, ARS was used to visualize scaffold calcification after 10 days in the three hydrogels. The staining confirmed that calcium deposition increased with the amount of nanofibers present (Figure 8), with mineralization was greatest for the 20% composite.

## 4. Conclusions

In this study, the osteogenic differentiation of ADSCs was investigated in photo-crosslinked HA hydrogels incorporating fragmented nanofibers. The addition of fragmented nanofibers to the hydrogel at fixed weight ratios of 10 wt.% and 20 wt.% not only increased the hydrogel modulus from 1762.5 to 2502.6 and 3122.5 Pa, respectively, but also led the cells to exhibit a more elongated and stretched morphology than with the hydrogel alone. The levels of the osteogenic biomarkers COL1, ALP and RUNX2 were significantly higher in the hydrogels containing nanofibers. Incorporating nanofibers at 20 wt.% increased the biomarker levels by 5-, 2.6-, and 5-fold, respectively, compared with their levels in the hydrogel without nanofibers. The COL1 protein expression findings concurred with those for mRNA expression, confirming osteogenic biomarker expression in the 20 wt.% composite system. Overall, this study demonstrated that the proposed fragmented nanofiber–hydrogel composite could potentially be applicable for bone tissue engineering due to its ability to support cell survival, growth and differentiation of stem cells into osteogenic differentiation.

## Figures and Tables

**Table 1:** The PCR primers used in this study.

Col1	F: TCCCTTTGGAGCACTTCTTATC R: CTTGGAGGCTGTTTCCTTACT
ALP	F: TGGAGTATGAGAGTGACGAGAA R: GGCTACCTTGTATCTCGGTTTG
RUNX2	F: CAGACAGAAGCTTGATGACTCTAA R: CGGGACACCTACTCTCATACT
GAPDH	F: CTCCTCACAGTTGCCATGTA R: GTTGAGCACAGGGTACTTTATTG

GAPDH indicates glyceraldehyde-3-phosphate-dehydrogenase. F and R indicate forward and reverse primers, respectively.
